# Supplementary material for: Pathophysiological Significance of Neutrophilic Transfer RNA-Derived Small RNAs in Asymptomatic Moyamoya Disease
Source: Cells. 2021 May 1;10(5):1086. doi: 10.3390/cells10051086 (PMC8147334; doi:10.3390/cells10051086)

2D Structure	Local AU	Position

```
2222                      2252
                       7mer-m8
5'-taGTATTGTTAAATTGTATA-AATATGTGGGt-3' utr
     | ||: |  ||::|: || | :|||||||
3'-ucCUUAGGA--UUGGCG-AUCUGGUACACCCu-5' tRF
                  1615 1413    765432
                  3' Pairing   Seed
```

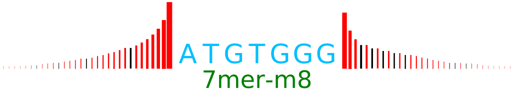

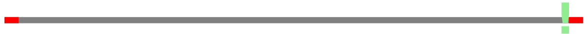

Supplement: Supplementary file 1 [file cells-10-01086-s001.zip › cells-1149161 3rd supplementary materials/Data Supplements/Data Supplement V.zip!/Data supplement 6-mRNAs targets of tsRNAs/tsRNA-Glu-TTC-010/Diagrams/tRF-Glu-TTC-010_vs_ENST00000368836(EEF1AKMT2).pdf]
